# Long-Lasting Follow-Up with Low-Dose Steroid in an 18-Year-Old Male with Rosai–Dorfman Disease

**DOI:** 10.1155/2020/5727569

**Published:** 2020-02-27

**Authors:** Volkan Karakuş, Yelda Morgül Dere, Dilek Ersil Soysal

**Affiliations:** ^1^Mugla Sıtkı Kocman University Training and Research Hospital, Department of Hematology, Mentese, Mugla 48100, Turkey; ^2^Mugla Sıtkı Kocman University Training and Research Hospital, Department of Pathology, Mentese, Mugla 48100, Turkey; ^3^İzmir University of Economics, Faculty of Medicine, Balçova, Izmir 35330, Turkey

## Abstract

Rosai–Dorfman disease (RDD) is a rare and benign pathology of sinus histiocytosis of unknown etiology. Lymphadenopathy is the predominant clinical manifestation, but diverse organs can also be affected. Histological features involve S-100+ histiocytes with characteristic nuclear features within the enlarged sinusoids of the lymph nodes. The clinical course is unpredictable, but is often benign with spontaneous resolution of disease in most patients. We report a patient with bilateral massive enlargement of cervical, axillary, and inguinal lymph nodes, moderately enlarged spleen, and a weight loss of 15 kg. Excisional biopsy from the cervical lymph node showed that the dilated sinusoids were infiltrated by lymphocytes, plasma cells, and large histiocytes with CD 68 and S-100 protein positive. Due to the slow progression of the disease, oral prednisolone with a body weight of 1 mg/kg was started in March 2016. The steroid dosage has been adjusted many times during the clinical follow-up. After 33 months, steroid treatment resulted in partial shrinkage of lymph nodes, the spleen returned to its normal size, and the patient gained weight. After 38 months of follow-up, no systemic symptoms, sign, or extranodal involvement were detected, and the patient continued with low-dose steroid treatment.

## 1. Introduction

Sinus histiocytosis with massive lymphadenopathy (SHML), also known as Rosai–Dorfman disease (RDD), was first described by Rosai et al. [1–4]. The disease is a benign histiocytic disorder with massive lymphadenopathy [2–5] and is usually self-limiting, with an unknown cause [2, 3]. Bilateral, painless, massive lymphadenopathy of the cervical lymph nodes is typical with the usual involvement of the other lymph nodes [2–5]. Cases with extranodal involvement [5–7] or disease limited to the skin has been reported [4]. Affected tissues demonstrate marked infiltration of plasmacytosis and of histiocytes that exhibit emperipolesis [7]. In most cases, accompanying laboratory findings include fever, leukocytosis, elevated erythrocyte sedimentation rate, and hypergammaglobulinemia [1–4]. The disease often shows a prolonged clinical course and is characterized with exacerbation and remission phases [7].

We present a sporadic case of an RDD patient followed for 38 months. The patient had nonspecific clinical symptoms and signs and normal laboratory and serological findings and was diagnosed by histopathological features from the lymph node biopsy specimen.

## 2. Case Presentation

An 18-year-old white man presented with a six-month-history of bilateral neck, axillary and inguinal lumps associated with fatigue, and weight loss of 15 kilograms. There was no history of fever, night sweats, rashes, nausea, vomiting, diarrhea, or change in appetite. His past medical history and family history were unremarkable. He had five years of experience of boxing. Physical examination revealed bilateral and multiple enlarged lymph nodes of various sizes between 25 × 10 mm, 24 × 8 mm, and 18 × 9 mm on the cervical, axillary, and inguinal regions, respectively, and a palpable splenomegaly below the left costal margin in deep inspiration. The size of the spleen measured by ultrasonography was 130 mm. The lymph nodes were soft, painless, and of massive proportions. No drain was noted from the lymph nodes, and the overlying skin was normal.

The complete blood count, erythrocyte sedimentation rate, serum C-reactive protein, serum albumin, and globulin and lactate dehydrogenase levels were within normal ranges. The peripheral blood smear showed normocytic and normochromic erythrocytes with polymorphonuclear leukocytes (PMNL) 55%, lymphocytes 40%, monocytes 4%, eosinophils 1%, and sufficient amounts of platelet clusters with absolute platelet count of 313 × 10^9^/L. Renal and liver function test results were unremarkable. Urine analysis was normal.

Serological markers for TORCH [8], Toxoplasmosis (T), Rubella (R), Cytomegalovirus (C), Herpes Simplex viruses (H), and Others (O), which included Hepatitis A, B, and C, Leptospirosis (not studied), Epstein Barr Virus (EBV), HIV, and Human Parvovirus B19, were negative.

**Figure 1:**
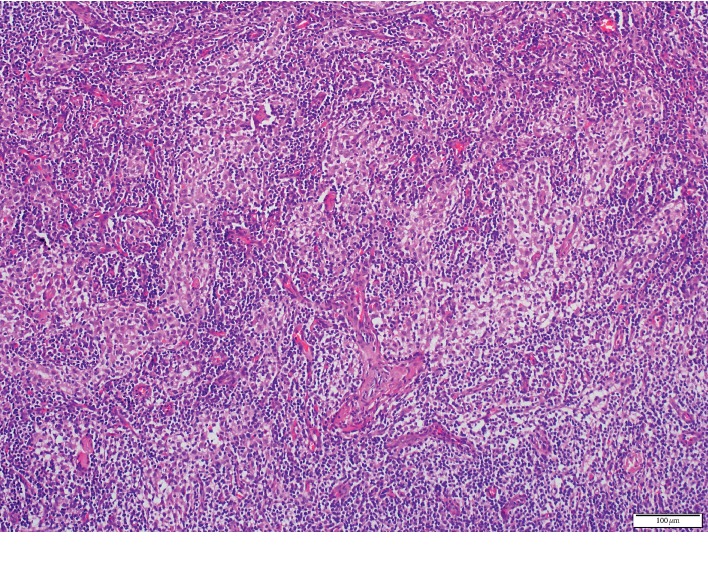
Cervical lymph node. Enlarged sinusoids filled with large histiocytes, HE, X40.

**Figure 2:**
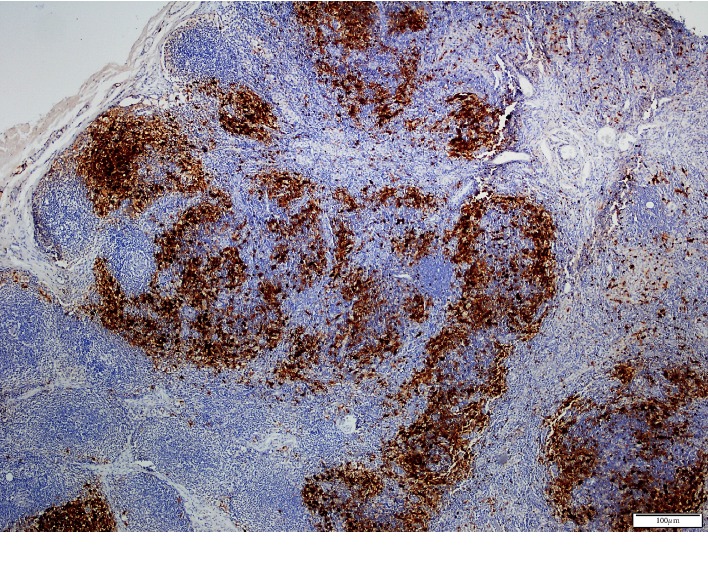
Cervical lymph node. S100 positivity, DAB, X40.

Computed tomography (CT) of the cranium and neck showed bilateral cervical lymph node enlargement, and CT of the thorax showed normal findings. Ultrasonography (USG) of the abdomen showed diffuse and moderately enlarged spleen. No abdominal lymphadenopathy or ascites were observed. Excisional biopsy of the cervical lymph node was performed. The histopathologic examination of the biopsy specimen demonstrated the CD 68 [+] large histiocytes within the enlarged sinusoids of the lymph node (Figure 1) and S-100 positivity (Figure 2). On the basis of histopathological diagnosis and considering the clinical condition of the patient, oral prednisolone 1 mg/kg/day was initiated. In the first month, steroid dose was reduced to 0.5 mg/kg/day and then to 0.3 mg/kg/day. This dose was maintained for 3 months and then gradually reduced to 0.1 mg/kg/day. After 33 months of follow-up, he was symptom-free. Weight loss and malaise resolved completely, and the spleen returned to its normal size, but cervical, axillary, and inguinal lymph nodes remained palpable with slight decrease in their size. By the 38th month, he was taking 4 mg low-dose oral prednisolone daily, and there was no recurrence of the symptoms or development of extranodal manifestations. Follow-up at regular intervals is being continued.

## 3. Discussion

Rosai et al. described the clinical manifestations of RDD over detailed analysis of 34 cases in 1972 [1, 6, 9]. They reported the first and second decades as the ages of onset [1, 10], the male sex was dominant, and SHML was associated to lower socioeconomic status [1]. In general, the disease affects children and adolescents [3, 4, 7, 9, 10], but it may also occur in older adults [4]; male and female proportions may vary [4, 7, 9], and different races are affected equally [9].

In patients with RDD, bilateral and painless cervical lymphadenopathy is the most prominent clinical manifestation [1–5, 9–12]. Axillary, inguinal, mediastinal, and retroperitoneal lymphadenopathy has been recorded to a minor degree [1, 2, 4]. Extranodal involvement, such as the skin [1, 3–5, 11], scalp, thyroid gland [12], palatine tonsil, soft tissues of the orbit, eyelid, and testicle [1, 3], rhinopharynx [9], bone and breast, central nervous system, and gastrointestinal tract, has been reported in patients with RDD [1, 3, 4]. Even though presentation with fever, night sweats, malaise, and weight loss is common in patients with RDD, they maintain a good general condition [1, 3, 4, 10]. Anemia, leukocytosis with neutrophilia, elevated erythrocyte sedimentation rate, and hyperglobulinemia are the dominating laboratory features [1, 3–5, 7, 9, 13]. Laboratory tests may involve bone marrow biopsy [1, 12] and serology in specific conditions [1, 3].

Many hypotheses have been developed to explain the unknown etiology of RDD. These include (a) invasion by bacteria such as Brucella and Klebsiella or invasion by viruses such as Epstein–Barr virus, herpes group viruses, and Parvovirus B19 [5–7]; (b) immune dysregulation with concomitant red blood cell autoantibodies [5, 7], arthritis, glomerulonephritis [7], autoimmune lymphoproliferative syndrome-1 [5], and lymphoma [2, 5, 13]; (c) aberrant response to unspecified antigens, such as HHV-6 [6, 12]; and (d) altered apoptosis by defective Fas/FasL signaling, which may trigger uncontrolled histiocytic proliferation [6]. In differential diagnosis, biochemistry results and serological and histological findings for possible etiologies did not support the presence of the microbiological agents, lymphoma, Langerhans cell disease, autoimmune lymphoproliferative syndrome-1, or of the immunologic disorders in our patient.

In SHML, the pathological aspect of the lymph node is characterized by sinusoidal dilatation infiltrated by the lymphocytes, plasma cells, and the histiocytes with large pale cytoplasms and vesicular nuclei [2, 3, 5]. Positive staining for S-100 and CD68 and negative staining for CD1a are the common immunohistochemistry findings [2, 5–7, 9, 10, 12]. Another diagnostic feature is the emperipolesis [1–4, 6, 9, 12], which shows phagocytosed lymphocytes and plasma cells within the histiocyte cytoplasm. In this patient, RDD was diagnosed by immunohistochemical examination of the lymph node biopsy specimen.

Data obtained from previous studies of RDD emphasized spontaneous regression and stable disease course [6, 11] with remission and exacerbation periods [6, 13]. The follow-up periods of the disease range from 2 months to 14 years [1]. Our patient has an average follow-up of 38 months, with good condition throughout his illness so far.

In the literature, there are different modalities recommended for the treatment of RDD [13]. Observation is the preferred choice of therapy in asymptomatic patients or if there is spontaneous resolution of adenopathies [10]. Reports have shown that treatment with steroids resolve fever and reduce lymph node size [1, 9, 10, 13], and the period to resolve clinical symptoms with corticosteroids ranges from 5 days to 6 months [13]. In our case, after 38 months of oral corticosteroid treatment, the symptoms disappeared completely, the spleen returned to its normal size, but the lymph nodes showed slight regression following disease diagnosis. However, in another case of Rosai–Dorfman disease with lymphadenopathy and cutaneous involvement, the clinical manifestations responded to low-dose oral prednisolone therapy within 3 months [11]. The same case remained in remission for 10 months and then showed a slight recurrence, which was overcome by an increase in prednisolone dose.

On the basis of the histological manifestations and the patient's clinical condition, oral prednisolone 1 mg/kg/day was initiated at diagnosis. Steroid dose was adjusted by reducing gradually from 1 mg/kg/day to 0.5 mg/kg/day, 0.3 mg/kg/day, and then to 0.1 mg/kg/day, respectively. After 33 months, weight loss and malaise resolved completely and the spleen returned to its normal size, but cervical, axillary, and inguinal lymph nodes remained palpable with only slight decrease in their size. Currently, after 38 months of follow-up, he is taking 4 mg low-dose oral prednisolone daily. We have observed no recurrence of the symptoms or extranodal involvement.

In some cases, mild decrease in the lymph nodes with radiotherapy was detected [1]. Chemotherapy or radiotherapy is recommended in patients with severe symptoms, and vital organ or system involvement [3, 6, 9, 12]. Surgical intervention is recommended for symptomatic, progressive, and surgically accessible lesions [3, 6, 11]. Studies have revealed the disease is usually self-limiting and rarely requires systemic therapy [3, 9, 11, 12]. This 18-year-old male patient presented with painless lymphadenopathy, weight loss, and fatigue for 6 months. It is not clear whether the disease will self-limiting or prednisolone will cure RDD or provide solely symptomatic relief. Long-term follow-up is necessary to recognize and prevent the recurrence of disease, and, if present, the underlying malignancy.

## 4. Conclusion

The morbidity and mortality of RDD is mostly related to the involvement of multiple extranodal regions in the disease [7]. In this case involving fatigue, weight loss, and enlarged multiple lymph nodes, treatment with prednisolone resulted in plateau-lasting disease stability in the patient. It will be remarkable if the patient initially unresponsive to steroid provides a good response to therapy later.
